# Influence of a multidirectional overground body weight support system on walking-related tasks in typically developing children and adolescents

**DOI:** 10.3389/fmed.2025.1690286

**Published:** 2025-11-21

**Authors:** Hubertus J. A. van Hedel, Sophia Rhiel, Emanuel Frei-Zuercher, Urs Keller

**Affiliations:** 1 Swiss Children's Rehab, University Children's Hospital Zurich, University of Zurich, Affoltern am Albis, Switzerland; 2 Children's Research Center, University Children's Hospital Zurich, University of Zurich, Zurich, Switzerland; 3 Department of Health Sciences and Technology, ETH Zurich, Zurich, Switzerland

**Keywords:** RYSEN, body weight support, walking, stand up and sit down, balance, transparency, spatiotemporal parameters, youth

## Abstract

**Introduction:**

The RYSEN is a multidirectional body weight support system for overground gait rehabilitation. It provides vertical unloading and enables unrestricted overground walking while preventing falls. While the RYSEN seems highly transparent in healthy adults, i.e., users only feel small forces from the device in response to their movements, we explored how the RYSEN affects the execution of various walking-related tasks in typically developing children and adolescents (TDCA).

**Methods:**

Nineteen TDCA, weighing 19.3–57.3 kg, participated. They performed several walking-related tasks in a randomized order using the RYSEN exercise modes: walking, stairs, stand up, sit down, and balance. They moved in the RYSEN with 20% body weight unloading. A 3D motion capture system collected movement parameters. Force plates recorded ground reaction forces. We compared various parameters between the RYSEN and no-RYSEN conditions and correlated the percentile differences in parameters between these conditions with the participants' body weight. Participants also completed several questions.

**Results:**

Walking: compared to the no-RYSEN condition, TDCA walked slower in the RYSEN, with shorter steps and greater step length variability (*p* ≤ 0.008). Along a curved path, TDCA walked with larger deviations (*p* ≤ 0.004). Stairs: participants walked slower and with wider steps when stepping on and off a higher-level surface (*p* < 0.001). Stand up and sit down: these tasks took longer with deviating movement patterns and ground reaction forces, particularly when sitting down (*p* < 0.001). During the dynamic balance tasks, the TDCA reached further forward and sideways in the RYSEN balance mode (*p* ≤ 0.003). Effect sizes were generally medium to large. Some correlation analyses, for example, those that included the percentile differences of walking speed and step width during straight walking, showed that lighter children were more affected than heavier children. The TDCA generally considered the RYSEN to facilitate most tasks.

**Conclusions:**

Therapists should be aware that each RYSEN exercise mode influences the performance of walking-related tasks differently and that some modes impact lighter children more than heavier children. Our next step is to evaluate the applicability of the RYSEN in the target population, i.e., children and adolescents with gait disorders.

## 1 Introduction

Children and adolescents with neuromotor disorders, such as cerebral palsy, may experience various sensorimotor and cognitive impairments that restrict their ability to control their posture, balance, and walking ability. Falls are also frequently reported in these individuals, with approximately 35% experiencing daily falls and about 30% reporting weekly or monthly falls (1). Consequently, therapists prioritize enhancing dynamic balance in pediatric neurorehabilitation. Due to the elevated fall risk in these patients, therapists remain near the patient to assist in case of imbalance. However, this close proximity complicates hands-off therapy and limits the patient's opportunity to practice dynamic balance tasks independently.

Over the past decade, tethered (i.e., cable-bound, being mounted to the ceiling) and untethered (i.e., battery-powered) technologies have been developed to complement conventional physiotherapeutic exercises. These devices enable multidirectional overground walking and walking-related tasks whilst providing body weight support and preventing falls (2). Such systems enable patients to safely walk around and complete tasks, navigate obstacles or overcome other challenges set by therapists.

However, these devices and systems have yet to be evaluated in children and adolescents. To our knowledge, only two studies have evaluated their applicability in pediatrics, both focusing on the untethered Andago (Hocoma AG, a DIH brand, Volketswil, Switzerland) (3, 4). The Andago is a powered wheeled frame with a dynamic body weight support, enabling users to move indoors on flat, firm, level surfaces but not walk sideways. It's easy to use, well-accepted by young patients with neurological gait impairments and therapists, and, compared to treadmill walking, allows for more natural stride-to-stride variability and inter-joint coordination (4). It also seems to require more attention and motor planning (3).

Tethered, multidirectional, overground, body weight support systems are mounted to the ceiling, while cables and a harness are connected to the user, allowing unobstructed walking in a restricted workspace. The FLOAT (Reha-Stim Medtec AG, Schlieren, Switzerland) (5), the RYSEN (Motek Medical, a DIH brand, Houten, The Netherlands) (6), and the relatively new ZeroG 3D (Aretech, Sterling, VA, USA) belong to this group of tethered multidirectional, overground, body weight support systems. Some advantages of these tethered systems compared to the Andago are that the user can move sideways, and it is much easier to turn around.

Importantly, these multidirectional, overground, body weight support systems need to provide sufficient support to enable successful walking, while empowering patients to perform their own movements without distortion, i.e., the systems should be transparent. In simple terms, transparency refers to the robot's ability to “get out of the way” (7, 8). For the RYSEN, this would mean that the user only feels small forces from the RYSEN in response to their movements.

While we are unaware of any studies that included the ZeroG 3D, the design of the FLOAT and its preliminary validation on healthy adults were presented in 2013 (5). The FLOAT has been studied for its transparency (9). The authors reported that the measured interaction forces of the FLOAT were small and varied between 3.3 N at low body weight support (52 N) to 17.6 N at high body weight support (376 N) when moving with constant velocity. The RYSEN was designed to have low power consumption, and perform at least as well as existing body weight support technologies in terms of human-robot interaction (6). One study investigated how the device transparency, support force vector direction, and harness attachment affected the gait pattern of healthy adults (10). The authors found that the device transparency along the rails' longitudinal direction was 40 times better than that of the FLOAT.

As these tethered systems have primarily been developed for and evaluated in adults, the various needs for the application of these technologies in children and adolescents may not be fully met (11). Indeed, we are unaware of studies investigating how these systems affect walking and walking-related tasks in children and adolescents. For several years, we have been using the RYSEN therapeutically in our rehabilitation clinic for children and adolescents with neurological disorders. As the largest patient group in our clinic is children and adolescents with cerebral palsy, this is also the largest group being treated in the RYSEN. Therapists train patients with severity grades ranging from Gross Motor Function Classification System level I to III, occasionally IV, in the RYSEN, as well as other patient groups with comparable functional levels. Despite the high transparency of the RYSEN, we have the impression that certain exercises are performed differently when children—particularly smaller, lighter ones—are inside the RYSEN compared to walking outside the system. However, we are aware that these differences may be caused, or at least confounded, by the physical impairments of these patients. Therefore, we decided that our first study should explore the influence of the RYSEN on various walking-related tasks in typically developing children and adolescents (TDCA). Understanding how TDCA adapt their movement execution when using such a technology could be valuable for therapists when interpreting movement tasks they observe in patients.

The RYSEN includes five exercise modes (i.e., *walking, stairs, stand up, sit down*, and *balance*) that we will explain later. By combining these exercise modes with different levels of body weight support and horizontal forces, a therapist can tailor the RYSEN settings to an individual patient. In this study, TDCA performed several walking-related tasks under different settings in the RYSEN and regular overground (no-RYSEN) conditions. Another manuscript in preparation focuses on the effects of different levels of body weight support (20 and 30%) and the application of horizontal forces in the *walking* exercise mode.

In this manuscript, we explore differences between the RYSEN and no-RYSEN conditions in TDCA. Specifically, we compare kinematics for (i) walking in a straight line and following a curved path, (ii) stepping on and off a higher-level surface, (iii) standing up and (iv) sitting down, and (v) reaching forward and sideways while maintaining balance. For (i), (iii), and (iv), we also compare ground reaction forces. In addition, we investigate whether differences in the parameters between the RYSEN and no-RYSEN conditions correlate with body weight.

## 2 Material and methods

### 2.1 Study design and general protocol

We designed a single-center, unblinded, experimental study to explore how TDCA performed several walking-related tasks with the RYSEN compared to walking without this technology. We invited TDCA to participate in a single measurement session between October and December 2021. The session lasted approximately 100 min.

We briefly welcomed the participants upon arrival and informed them of the protocol and tasks. We measured body weight and height with shoes to anticipate future projects, where we will include children and adolescents with neuromotor disorders who often depend on wearing (orthopedic) shoes. We selected the appropriate harness size to ensure proper vertical unloading and attached reflective markers to the participant's shoes, hands, shoulders, and the RYSEN sling bar for kinematic recordings.

The participants performed various walking-related tasks in RYSEN and no-RYSEN conditions. The order of the conditions was randomized. Before the condition with the RYSEN, the participants became acquainted with the RYSEN in a short familiarization session lasting several minutes. The walking-related tasks were clustered into the RYSEN exercise modes: *walking, stairs, stand up* and *sit down*, and *balance*. The order of these clusters was randomized between participants and the two conditions. During the measurements, we projected lines on the floor for guiding the walking tasks (*walking, stairs*) and squares indicating foot placement (*stand up, sit down*, and *balance*) to facilitate task execution (e.g., walking in a straight or curved path). A short break between tasks was possible. Furthermore, the participants responded to a questionnaire designed for the study to rate their subjective perceptions.

We also offered the opportunity to play a short exergame projected on the floor to keep the participants motivated. It was played at the end of the session. We further noted any technical issues and adverse events. Finally, the reflective markers were removed, and a small gift was given.

### 2.2 Participants

We recruited TDCA aged 5–18 years, at least 100 cm tall (in line with company specifications), with no known neurological, cardiovascular, or musculoskeletal diagnoses. The participants were children of employees of our clinic or acquaintances. We hypothesized that the RYSEN might affect the performance of walking-related tasks more in participants with a lower body weight and less in those with a higher body weight. Therefore, we used a purposive sampling strategy to evenly distribute the targeted 15–20 participants across three weight categories: 17–25 kg, 25–45 kg, and over 45 kg. Participants in the first group wore a special RYSEN harness featuring additional weight (total weight harness about 3 kg) to achieve the necessary system rope pretension for a good RYSEN performance. We selected the 45 kg threshold because a 13-year-old child weighs 46 kg on average, and this weight also reflects the lower 3% percentile of 16-year-old healthy males and the lower 3%-percentile of 18-year-old females (12). Participants should also be able to understand simple instructions, express pain or discomfort, and see projections on the floor.

Parents and children were informed verbally and in writing. Participants and their parents were given sufficient time to consider participation. Children between 10 and 14 years received easy-to-understand written information. While all participants and their parents had to give verbal consent, parents and participants older than 14 years received a detailed written information sheet and had to provide written informed consent. The Ethics Committee of the Canton of Zurich confirmed that this study does not fall under the Human Research Act (BASEC-Nr. Req-2021-00848).

### 2.3 The RYSEN

**Figure 1:**
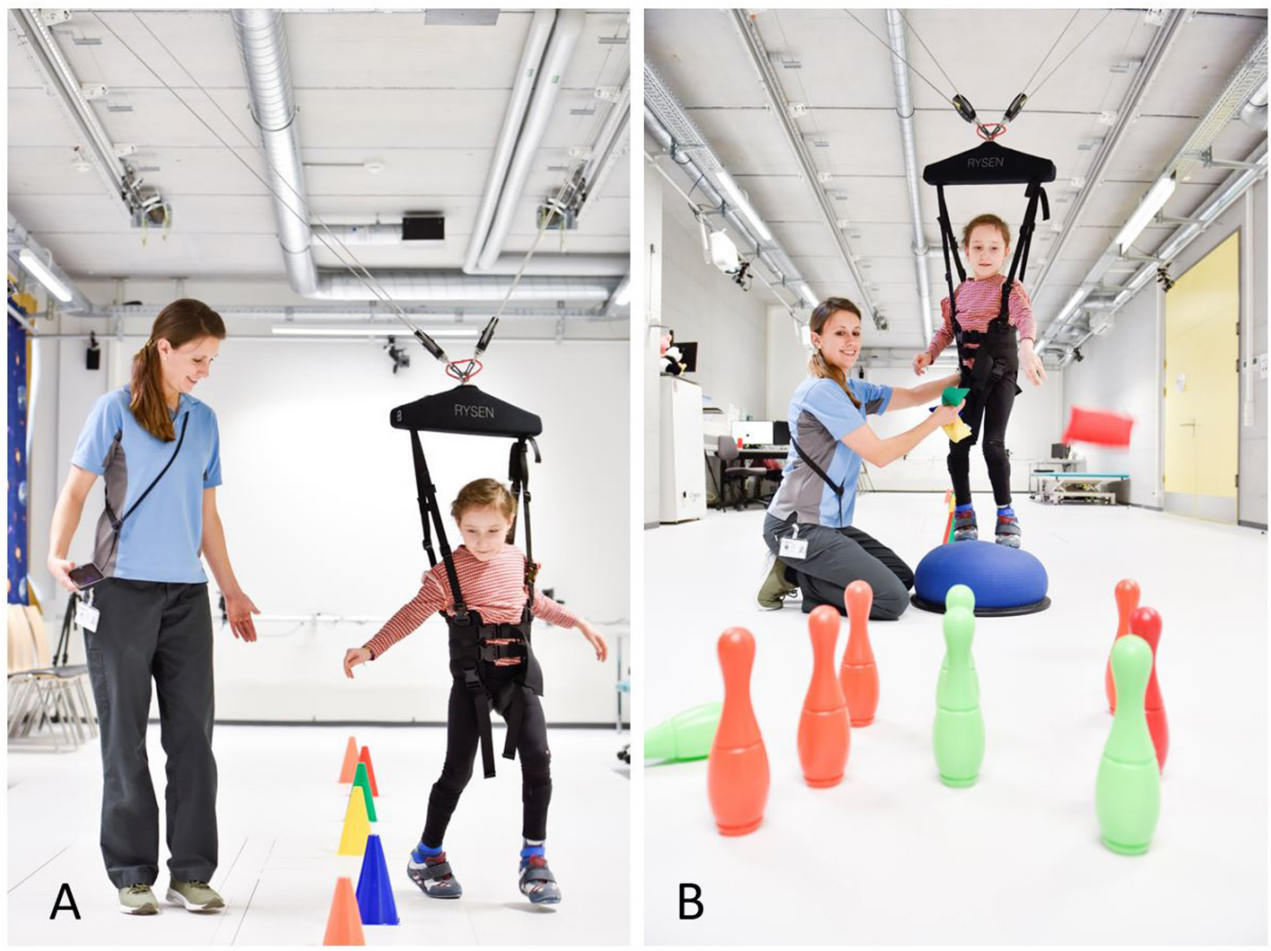
Clinical use of the RYSEN. Young patient walking secured and with body weight unloading in the RYSEN while **(A)** walking and negotiating obstacles at about 20% body weight unloading in the *walking* mode or **(B)** balancing on a wobbly uneven surface while trying to overthrow the bowling pins with a sandbag using the *balance* mode. Picture with permission from the University Children's Hospital Zurich (Copyright © University Children's Hospital Zurich). With kind permission from the child and its parents.

The RYSEN (Motek Medical BV, a DIH company, Houten, Netherlands) is a multidirectional body weight support system. It consists of a harness worn by the patient, which connects to the RYSEN sling bar, enabling safe overground walking (Figure 1). Motor units are mounted to the ceiling. They control the cable-suspended sling bar through rails and pulleys. The five different-sized geared motors and the kinematics of the RYSEN allow for decoupling different degrees of freedom. The pulleys on the sling bar enable a certain passive movement. The combination of these parts results in a high system transparency, particularly in the longitudinal direction, where the patient only feels a low resistance when walking back and forth.

For adults, the vertical forces, i.e., the body weight support, can be manually adjusted between 10 and 60% of the body weight. Lightweight children might not be trained at very low body weight unloading levels because the system needs about 4 kg rope tension for good performance (e.g., a child weighing 20 kg cannot be trained with 10% body weight unloading because the system rope tension would be 2 kg). In addition, a force ranging from −5 to +7% of the body weight can support or resist the movement in the anteroposterior direction. The workspace width depends on the height of the room. In our center, the RYSEN allows unrestricted overground walking in a workspace of approximately 10.0 × 1.3 meters.

**Figure 2:**
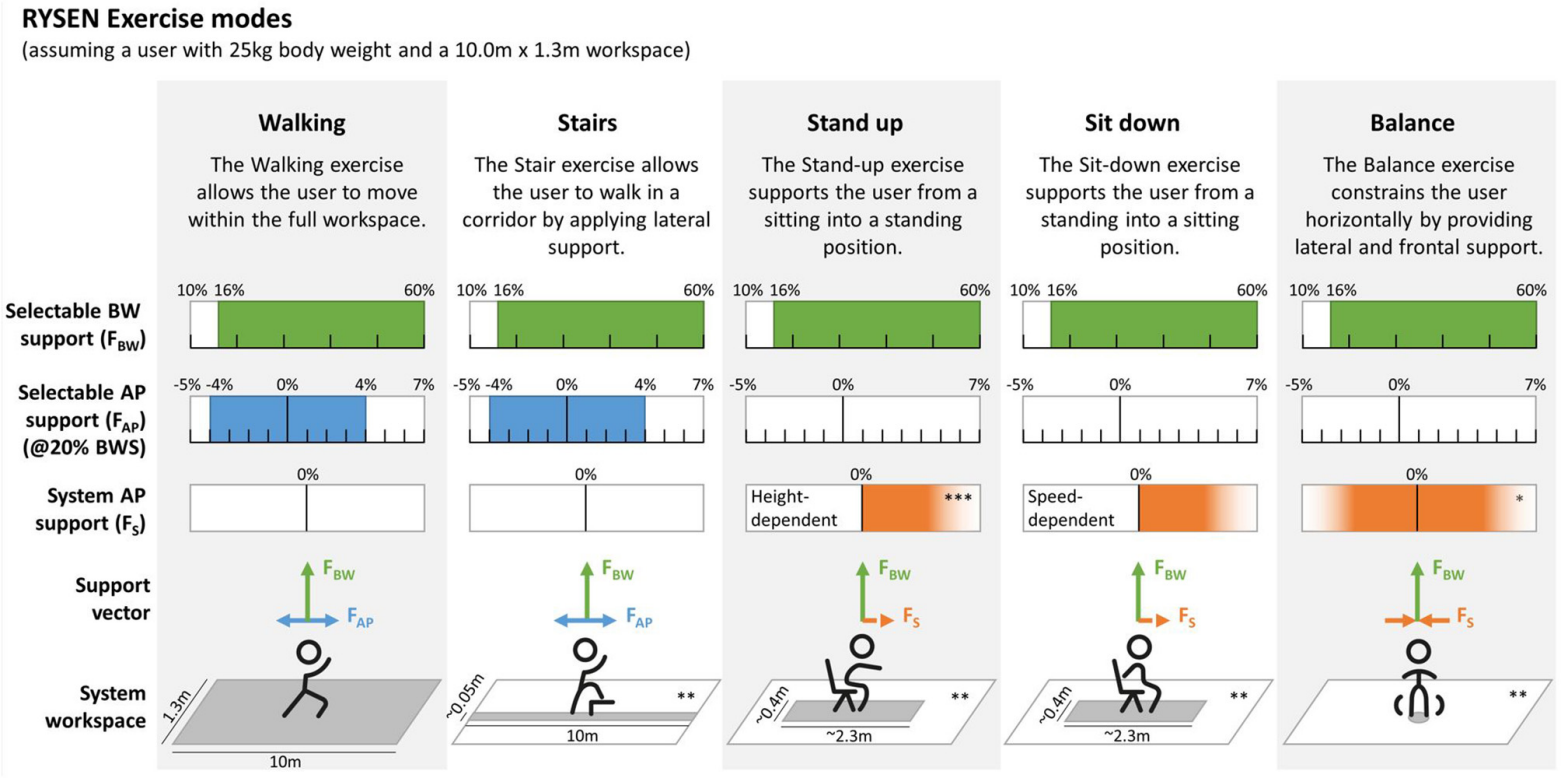
Exercise modes of the RYSEN. Shown are the various exercise modes implemented in the RYSEN with a short description, the level of body weight (BW) support that can be selected (green), the forces that the therapist can select to assist or resist in anteroposterior (AP) direction (blue), the support in AP direction provided by the system (orange), and the workspace (dark gray), assuming a user weighing 25 kg. The user's weight of 25 kg limits the full range of body weight support (green) or anteroposterior forces (green) that can be selected.* The stiffness of the haptic walls limiting the workspace can be seen as supportive forces to keep the user in a given location. ** The location of the workspace depends on the user's location when the exercise starts. *** The anterior support force depends on the user's shoulder height during stand-up, creating a forward force to get out of the chair, which reduces to zero when the user stands.

The RYSEN has five exercise modes. Each mode enables walking-related tasks with predefined and adjustable parameters (Figure 2). *Walking* enables unrestricted walking within the workspace. At the same time, vertical and horizontal forces can be applied to partially unload in the vertical direction and support or resist in the longitudinal direction of the system. *Stairs* enables practicing walking up or down up to 30 cm high obstacles or stairs (i.e., about two staircase steps). Lateral forces restrict the workspace in the mediolateral direction to a minimum to prevent falling sideways. This corridor is approximately 5 cm narrow at the height of the sling bar. Vertical forces enabling body weight unloading and anteroposterior forces supporting or resisting the movements can be applied. *Stand up* and *sit down* support by providing body weight unloading and horizontal forces in the forward direction, i.e., supporting standing-up or slowing down the sitting-down movement by increasing the resistance. Finally, in *balance*, the RYSEN constrains in the mediolateral and anteroposterior direction while body weight unloading can be provided.

### 2.4 3D motion analysis and recording of ground reaction forces

Measurements were performed in the gait laboratory of the Swiss Children's Rehab clinic. Spatiotemporal motion characteristics were recorded with 12 Vicon Vero 2.2 megapixel high-speed cameras (Vicon Motion Systems, Oxford, UK) at 100 Hz. Fifteen 16 mm diameter self-adhesive reflective markers were placed at specific anatomical landmarks. Five markers were placed on each shoe: (1) first metatarsophalangeal joint, (2) distal phalanx of the second toe, (3) fifth metatarsophalangeal joint, (4) lateral malleolus, and (5) heel. The markers on the distal phalanx of the second toe and the heel were positioned at the same height. We also placed one marker on each hand (third metacarpophalangeal joint) and acromioclavicular joint, and one centered on the right scapula. The trials were recorded and labeled in the Vicon Nexus software (V2.7, Vicon Inc., Oxford, UK) and exported to MATLAB (V R2020a, The MathWorks Inc., Natick, MA, US) for further processing.

Two 50.8 cm × 45.4 cm × 11.0 cm AMTI biomechanical multiaxial force plates (AMTI Inc., Waterdown MA, USA) embedded in the floor were used to record the ground reaction forces. Data recordings from the Vicon system and the force plates were synchronized. In MATLAB, the recordings were imported and automatically routed to different evaluation pipelines based on the RYSEN exercise modes to compute the spatiotemporal and kinetic parameters.

### 2.5 Walking-related tasks and their outcome parameters

The participants performed the walking-related tasks with 20% body weight unloading because this is the level of body weight support most frequently used by the therapists in our clinic. Twenty percent unloading is also the minimum level of support that can be set for patients with a body weight of 20 kg (or 17 kg body weight and the 3 kg harness) to achieve the minimally required 4 kg pretension. Furthermore, we did not apply any additional horizontal forces besides the horizontal forces included in some of the RYSEN exercise modes [like *stand up*, see also Figure 2 system anterior-posterior (AP) support]. To balance collecting sufficient data for reliable results with preventing participants from losing interest, concentration, or becoming fatigued, we individually selected the number of repetitions for each task, depending on the parameter to be calculated.

**Figure 3:**
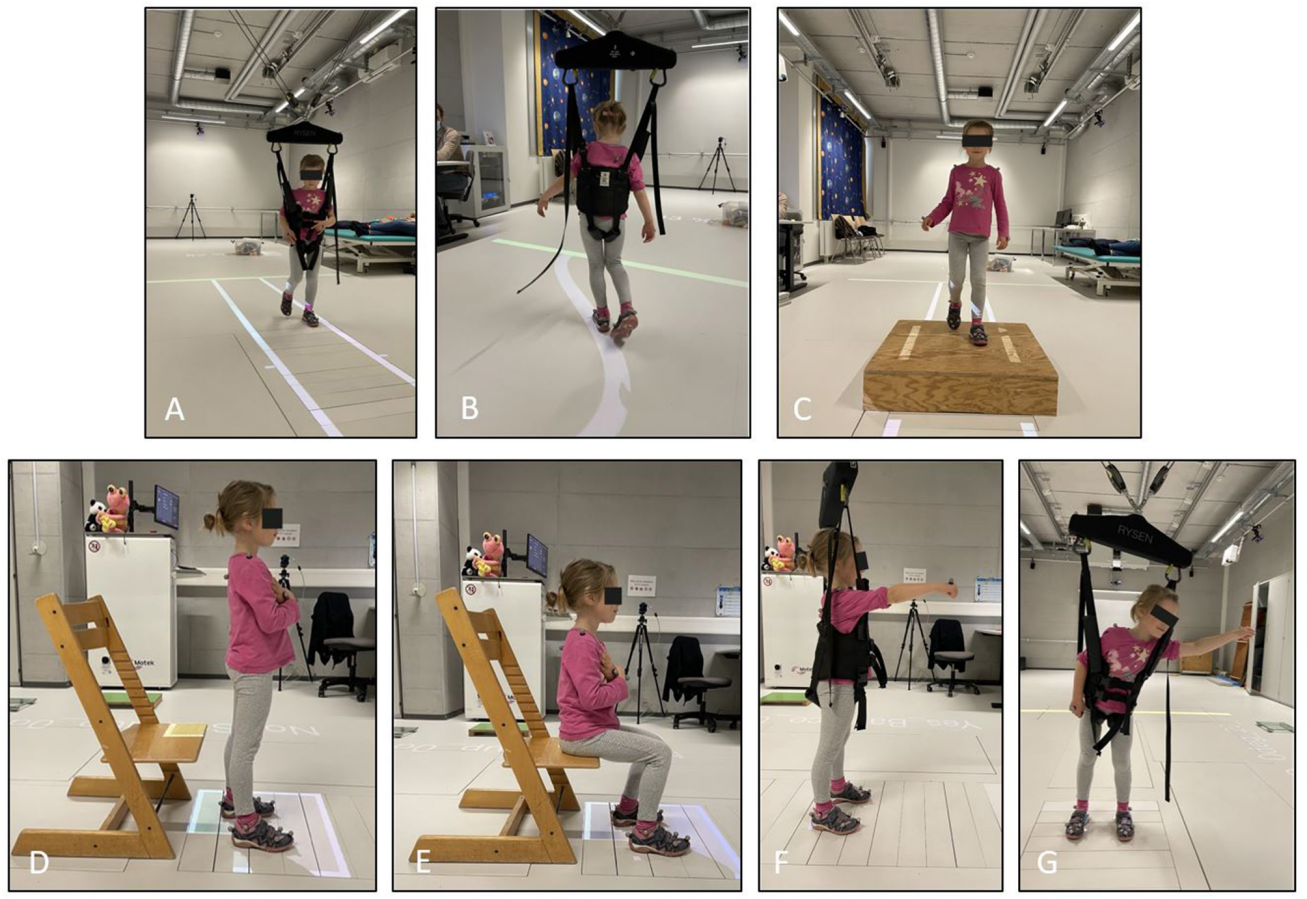
Illustrations of the tasks. **(A)** Walking between the projected straight lines in *walking* mode. **(B)** walking as precisely as possible on the curved line projected on the floor with three synchronized beamers under RYSEN conditions in *walking* mode. **(C)** stepping on and off the higher-level surface in the no-RYSEN condition. **(D, E)** Standing up and sitting down in no-RYSEN conditions. Finally, **(F)** reaching forward, and **(G)** reaching sideways in RYSEN *balance* exercise mode. Picture with kind permission from the child and its parents.

We investigated two walking tasks in the RYSEN *walking* exercise mode. When walking along a straight path (Figure 3A), we asked each participant to walk straight forward at their preferred walking speed. Participants completed several trials (approximately seven) per condition (RYSEN and no-RYSEN) until at least three valid ground reaction recordings were obtained. The inner width between the projected lines was 45 cm. We calculated the foot center positions at each stance phase as the midpoint between the second toe and heel markers. These positions were used to calculate the following parameters for each participant over all the recordings of the walking trials: average step length, measured from the center of one foot to the center of the other foot in the walking direction (m); step length variability, i.e., the standard deviation of step length (m); average step width, i.e., measured between the center of one foot to the center of the other foot, perpendicular to the walking direction (m); and average walking speed (ms^−1^).

When walking along a curved path (Figure 3B), participants were instructed to walk at their preferred speed over a 10 cm wide curved line projected on the floor as accurately as possible. Participants completed four trials per condition. In addition to the parameters mentioned above, we calculated for each participant the average foot deviation from the projected line on the floor, which was calculated as the average orthogonal distance between the foot center positions and the midline of the projected path over all the curved trials (mm).

In the RYSEN *stairs* exercise mode, we investigated ascending and descending a higher-level surface (i.e., a 17 cm high and 90 × 90 cm wide and long wooden box; see Figure 3C). Participants repeated this task four times for each condition. The inner width between the projected lines was, again, 45 cm. The outcome parameters were the average walking speed while crossing the higher-level surface (ms^−1^), calculated for each participant between two strides before and two after the wooden box, and the step width (m).

The RYSEN *stand up* and *sit down* exercise modes were tested in alternating order. *Stand up*: the participant sat on a height-adjustable chair with the hip and knee joints flexed at 90°. The feet were placed on the force plate. The arms were crossed in front of the chest. We then asked the participant to stand up (Figure 3D). In *stand up* mode, the RYSEN provides an anterior assisting force in addition to the body weight support. *Sit down*: the participant stood on the force plate with the feet shoulder-width apart and was instructed to sit down (arms again crossed in front of the chest; see Figure 3E). In *sit down* mode, the RYSEN provides increased resistance to decelerate downward movement. The standing-up and sitting-down tasks were repeated three times for each condition.

We evaluated three outcome parameters for standing up and sitting down. First, we calculated a distance-path ratio. We determined the midpoint between the left and right acromioclavicular markers and calculated the sagittal movement from start to end separately for standing up and sitting down. We then normalized the start position in a coordinate plane to 1000, 1000 and calculated the length of each path to the end of the movement. We also calculated the direct distance between the start (1000, 1000) and the endpoint. For each of the three participant trials per condition, we calculated the length for the movement path, the direct distance, and the according distance-path ratio by dividing the path length by the direct distance. Finally, the resulting distance-path ratio per condition was computed by taking the mean of all three trials. A distance-path ratio of 1 would indicate that the movement path perfectly followed the direct distance. Second, we calculated the duration of standing up and sitting down (s) from the movement initiation timepoint to the balanced standing and sitting positions, respectively. Third, we examined the ground reaction forces. To account for the difference in ground reaction amplitudes between partially unloaded (RYSEN) and regular (no-RYSEN) conditions, we normalized the vertical force values with the not-unloaded body weight to obtain a value of 1 when standing statically and unloaded in both the RYSEN and the no-RYSEN condition.

Finally, we investigated three tasks in the RYSEN *balance* exercise mode, i.e., reaching forward and reaching sideways left and right (cm). For these tasks, the participant stood facing the longitudinal direction of the RYSEN and with their feet at shoulder width (controlled by participant-specific floor projections). In reaching forward, i.e., a functional reach test in anterior direction (13), we asked the participant to move the outstretched arm forward as far as possible without moving the feet or falling (Figure 3F). In reaching sideways, i.e., a lateral functional reach test (14), we asked the participant to move their outstretched arm as far as possible to the left or right side, respectively, without moving the feet or falling (Figure 3G). Each participant performed three trials of each task in each condition, and we calculated the average reaching distance (in cm) for each condition and direction per participant. Because the RYSEN *balance* mode severely restricts the participant, each participant repeated the reaching forward and reaching sideways tasks in the RYSEN *walking* exercise mode. Thus, while we compared the RYSEN to the non-RYSEN condition for each task, we compared the reaching forward and sideways tasks in the no-RYSEN condition to two RYSEN conditions, namely the RYSEN *balance* and *walking* exercise modes.

### 2.6 Questions

After each task, the participants scored whether the RYSEN had hindered or helped them using a visual analog scale (hindered a lot = 0; helped a lot = 10). At the beginning and end of the measurement session, we asked whether the harness was comfortable (0 = very uncomfortable; 10 = very comfortable). At the end, we asked whether the measurement session had been fun (0 = no fun at all; 10 = a lot of fun).

### 2.7 Statistical analyses

We used RStudio version 1.3.959 (R Foundation for Statistical Computing, Vienna, AUT) and SPSS version 27 (IBM, Armonk, NY, USA). Data distribution was tested using the Shapiro-Wilk test. Differences between the conditions, i.e., RYSEN vs. no-RYSEN, were compared statistically using paired *t*-tests or non-parametric Wilcoxon signed-rank tests if the data were not normally distributed. As participants performed the reaching forward and reaching sideways tasks in three different conditions/exercise modes, we applied a repeated measures analysis of variance or Friedman's test if the data were not normally distributed. In case of statistically significant differences, we performed consecutive *post-hoc* tests using paired *t*-tests or the non-parametric Wilcoxon signed-rank tests. While α was generally set at 0.05, we adjusted it to 0.0167 for these latter pair-wise evaluations. As this was more an explorative study, we presented effect sizes accompanying the statistical analyses. For a normal distribution, we calculated Cohen's *d* (mean difference/SD differences), with *d* exceeding 0.2, 0.5, or 0.8 representing a small, medium, or large effect size, respectively. For data that were not normally distributed, the *r*_*ES*_ (z score/√N) was calculated, with values exceeding 0.1, 0.3, or 0.5 representing a small, medium, or large effect size, respectively.

To investigate whether the RYSEN influenced particularly the children who weighed less during the walking-related tasks, we subtracted for each parameter the RYSEN from the no-RYSEN conditions, expressed the difference as a percentage of the no-RYSEN condition, and correlated these percentages with the participants' body weight. Due to the relative small sample size, we used Spearman's (ρ) correlation analysis and applied bootstrapping with 2,000 samples using the Bias-corrected and accelerated (BCa) method to calculate its 95% confidence interval (95%CI). We interpreted the magnitude of the correlation coefficient as follows: 0–0.25 (no or little relationship), 0.25–0.50 (fair degree), 0.50–0.75 (moderate to strong relationship), 0.75–1.00 (very strong to excellent).

Finally, the responses to the visual analog scale questions were described and also compared between the conditions using a repeated measures analysis of variance or a Friedman's test, depending on the data distribution.

## 3 Results

### 3.1 Participants

The participants (nine females, ten males) were 10.1 ± 3.5 years old (mean ± SD), 141.9 ± 19.3 cm tall, and weighed 35.8 ± 12.7 kg. Five participants weighed less than 25 kg group (age: 6.3 ± 1.0 years, weight: 22.7 ± 2.3 kg, height: 120.0 ± 5.8 cm), 8 between 25 and 45 kg (age: 9.3 ± 1.5 years, weight: 31.9 ± 6.2 kg, height: 138.4 ± 9.9 cm), and 6 weighed more than 45 kg (age: 14.4 ± 1.5 years, weight: 51.9 ± 4.0 kg, height: 164.7 ± 7.1 cm). Six participants wore the XS size harness (one participant required the XS harness despite having a body weight of 25 kg), 10 wore the S size, and three wore the M size. Twelve participants reported that their right leg was the dominant leg, i.e., the foot used to kick a ball.

### 3.2 Walking along a straight path

**Figure 4:**
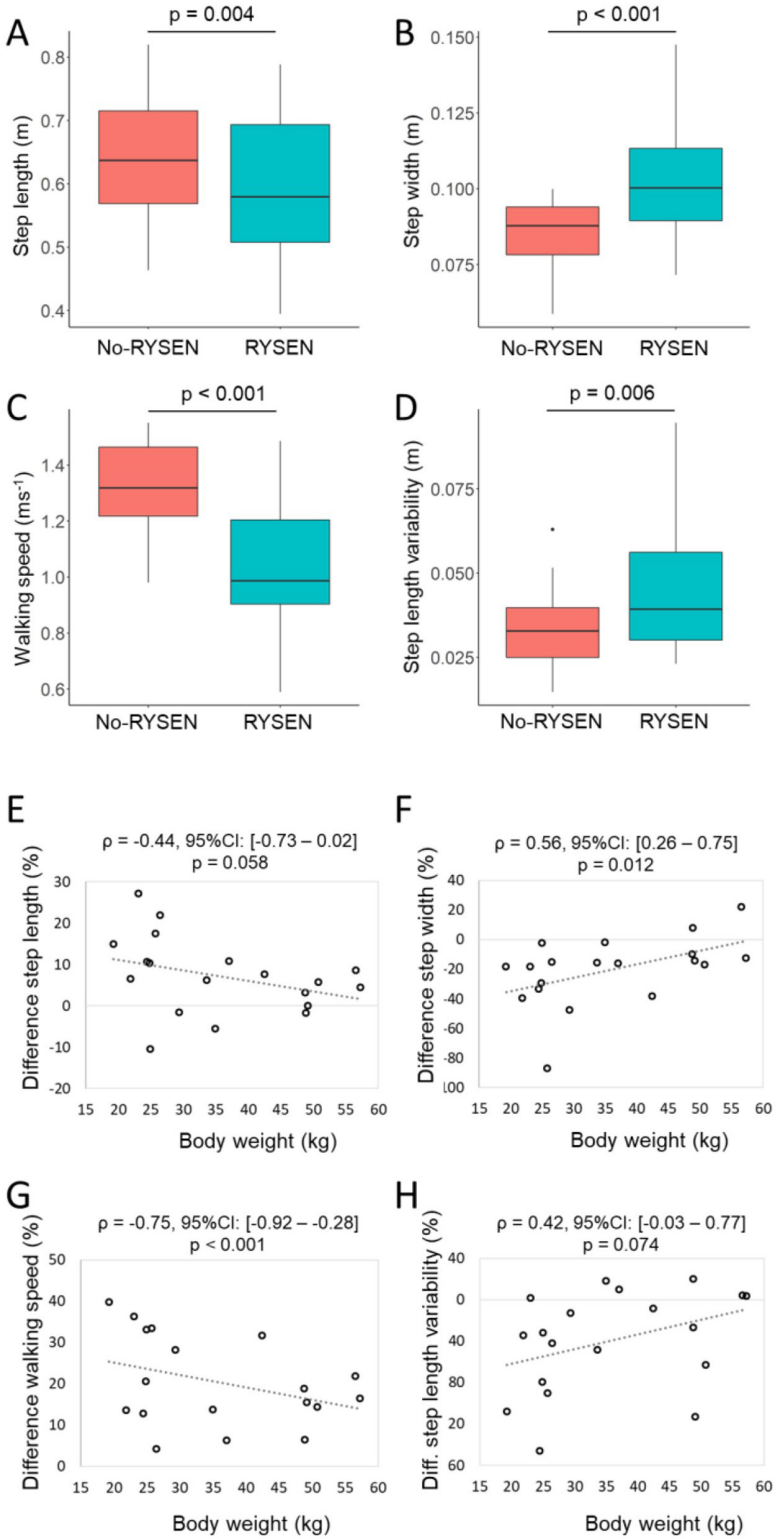
Gait characteristics during straight walking in the RYSEN and no-RYSEN conditions and body weight correlation analyses. **Top**: box and whisker plots showing differences in gait characteristics when walking in the RYSEN (turquoise) and no-RYSEN (red) conditions for **(A)** step length, **(B)** step width, **(C)** walking speed, and **(D)** step length variability. **Below**: correlation analyses between the body weight of the participants and the percentual differences in **(E)** step length, **(F)** step width, **(G)** walking speed, and **(H)** step length variability between walking with and without RYSEN. The dotted line indicates the linear trend. ρ, Spearman's correlation coefficient; 95%CI, 95% confidence interval of the correlation coefficient; *p, p*-value.

Compared to walking straight forward in the no-RYSEN condition, the participants reduced their step length and walking speed in the RYSEN, while their step width and variability in step length increased significantly (Figures 4A–D, Table 1). When analyzing the correlations between body weight and the differences in parameters [(RYSEN minus no-RYSEN)/no-RYSEN^*^100] (Figures 4E–H), we found a moderate and strong relationship between body weight and step width and walking speed, respectively, indicating that the lighter the participant, the larger the increase in step width and reduction in walking speed compared to the no-RYSEN condition. For the other parameters, the correlations were of fair magnitude and approached significance.

**Table 1:** Differences in outcome parameters between no-RYSEN and RYSEN conditions.

**Task**	**Outcome**	**No-RYSEN**	**RYSEN**	***p*-Value**	**Cohen's *d***
Straight walking	Step length (m)	0.64 ± 0.09	0.60 ± 0.12	0.004	0.75
	Walking speed (ms^−1^)	1.32 ± 0.17	1.04 ± 0.24	< 0.001	1.88
	Step width (m)	0.08 ± 0.01	0.10 ± 0.02	< 0.001	0.94
	Step length var. (m)	0.03 ± 0.01	0.04 ± 0.02	0.006	0.72
Curved walking	Step length (m)	0.51 ± 0.09	0.45 ± 0.10	< 0.001	1.22
	Walking speed (ms^−1^)	0.82 ± 0.21	0.59 ± 0.16	< 0.001	1.17
	Foot deviation (mm)	16.6 ± 4.0	24.8 ± 11.6	0.004	0.76
	Step length var. (m)	0.05 ± 0.01	0.08 ± 0.03	0.001	0.87
Stairs	Step width (m)	0.085 ± 0.013	0.115 ± 0.027	< 0.001	1.15
	Walking speed (ms^−1^)	1.16 ± 0.20	0.74 ± 0.16	< 0.001	2.83
Stand up	Duration (s)	1.73 ± 0.39	2.55 ± 0.58	< 0.001	1.36
	Distance-path ratio ()	1.72 ± 0.12	1.50 ± 0.20	< 0.001	0.99
Sit down	Duration (s)	3.31 ± 0.70	4.55 ± 1.15	< 0.001	1.81
	Distance-path ratio ()	1.88 ± 0.20	1.13 ± 0.13	< 0.001	3.72
Reaching (balance mode)	Frontal (cm)	25.8 ± 6.3	31.0 ± 8.6	0.002	0.82
	Left (cm)	18.4 ± 4.7	26.7 ± 6.5	< 0.001	1.54
	Right (cm)	19.9 ± 6.9	27.6 ± 8.1	< 0.001	1.05
Reaching (walking mode)	Frontal (cm)	25.8 ± 6.3	25.1 ± 6.8	0.62	0.12
	Left (cm)	18.4 ± 4.7	17.8 ± 4.8	0.59	0.13
	Right (cm)	19.9 ± 6.9	22.9 ± 5.0	0.014	0.63

step length var., step length variability.

### 3.3 Walking along a curved path

Compared to walking in the no-RYSEN condition, participants reduced their step length and walking speed, while they increased the foot deviation from the projected curved line and the step length variability (Table 1). Correlations between the body weight of the participants and the differences in the parameters were fair for the step length [ρ = −0.36, *p* = 0.14, 95%CI: (−0.66 to −0.07)] and absent for walking speed [ρ = 0.01, *p* = 0.96, 95%CI: (−0.47 to 0.50)] but very strong for the deviation [ρ = 0.80, *p* < 0.001; 95%CI: (0.65–0.87)] and step length variability [ρ = 0.80, *p* < 0.001, 95%CI: (0.62–0.89)].

### 3.4 Stairs

When stepping on and off the higher-level surface, participants increased their step width with the RYSEN while reducing their walking speed (Table 1). Correlations between the body weight of the participants and the differences in parameters were strong for the step width [ρ = 0.62, *p* = 0.004, 95%CI: (0.13–0.87)] and fair for walking speed [ρ = −0.32, *p* = 0.18, 95%CI: (−0.84 to 0.31)].

### 3.5 Stand up and sit down

**Figure 5:**
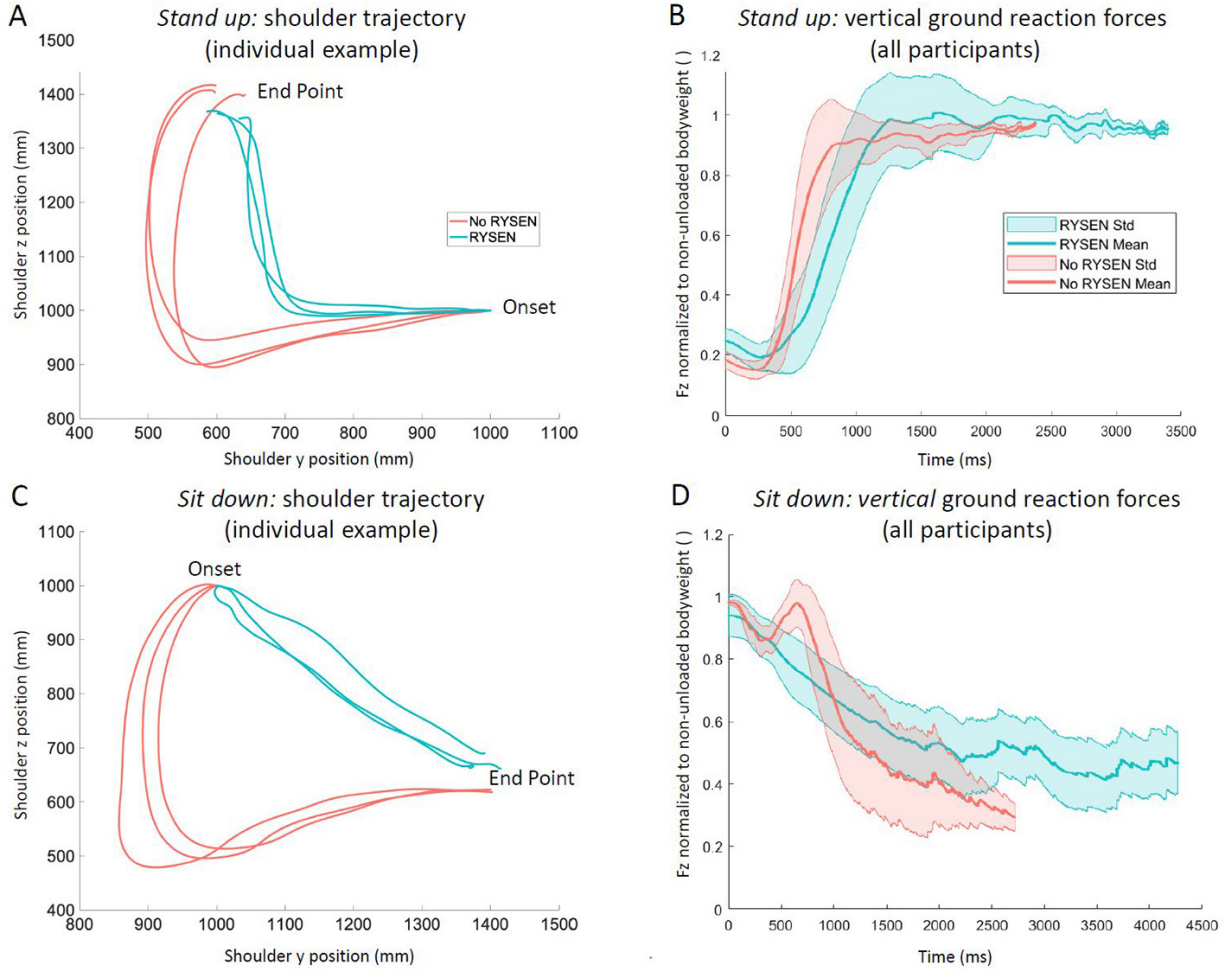
Motion kinematics and vertical ground reaction forces during *stand up* and *sit down*. **(A, C)** Individual examples of the kinematics of the midpoint between the two acromioclavicular joint markers during the three trials of **(A)** standing up and **(C)** sitting down (with RYSEN: turquoise; no-RYSEN: red). **(A)** When standing up without RYSEN, the participant moves initially forward and shortly downward with the shoulder before the shoulder rises. In contrast, in the RYSEN, the participant moves shortly forward with the shoulders and then up. **(C)** When sitting down, the participant moves shortly forward and down before the shoulder moves backward and upward. In the RYSEN, a completely different, very linear movement is performed. **(B, D)** Vertical ground reaction forces averaged over all participants for the RYSEN (turquoise) and no-RYSEN (red) conditions. We subtracted 20% of the body weight in the no-RYSEN condition to create equal amplitudes to simplify the visual interpretation of the ground reaction force patterns. While the pattern of the ground reaction forces for standing up appear similar in shape, despite being performed quicker in the RYSEN, the patterns are very different in the sitting down condition. Fz, vertical ground reaction force; Std, standard deviation.

Standing up from the chair with the RYSEN took longer, while the distance-path ratio was much smaller, indicating a more linear movement of the shoulders between the start and end points (Table 1). To understand how the participants stood up in the RYSEN, please refer to Figure 5A, where we show the mean movement trajectories of the midpoint between the acromioclavicular markers of the three trials of an individual participant. While the pattern of the vertical ground reaction forces is somewhat comparable between the RYSEN and no-RYSEN conditions, it clearly shows the longer time taken by the participant to stand up in the RYSEN (Figure 5B). Correlations between the body weight of the participants and the differences in parameters were little to fair [time needed to stand up: ρ = 0.12, *p* = 0.64, 95%CI: (−0.35 to 0.54); distance-path ratio: ρ = 0.26, *p* = 0.29, 95%CI: (−0.25 to 0.66)].

The differences between the conditions were more pronounced for sitting down. Sitting down took longer in the RYSEN condition, while the distance-path ratio was much shorter (see Figure 5C and Table 1). The distance-path ratio approached almost 1.0 in the RYSEN condition, indicating that the movement approached a straight linear shoulder movement trajectory between the start and end points. The patterns of the vertical ground reaction forces differed largely between the two conditions (Figure 5D). The correlations between the body weight of the participants and the differences in the parameters were little to fair [time needed to sit down: ρ = −0.22, *p* = 0.36, 95%CI: (−0.57 to 0.24); distance-path ratio: ρ = −0.35, *p* = 0.14, 95%CI: (−0.77 to 0.23)].

### 3.6 Balance

The functional reach tests in frontal and lateral directions were performed in the no-RYSEN condition and in the RYSEN condition in the *walking* and *balance* modes. We found significant differences between these conditions/modes for reaching forward [*F*(2, 17) = 16.2, *p* < 0.001], to the left [*F*(2, 17) = 22.1, *p* < 0.001], and right [*F*(2, 17) = 10.0, *p* = 0.001]. Pair-wise comparisons showed that the participants reached significantly further in the RYSEN condition under the *balance* mode.

In the RYSEN *balance* mode, participants reached significantly further in the frontal (31.0 ± 8.6 cm), left (26.7 ± 6.5 cm), and right (27.6 ± 8.1 cm) directions compared to both the *walking* mode (frontal: 25.1 ± 6.8 cm, *p* < 0.001, *d* = 1.33; left: 17.8 ± 4.8 cm, *p* < 0.001, *d* = 1.38; right: 22.9 ± 5.0 cm, *p* = 0.003, *d* = 0.78) and the no-RYSEN condition (see Table 1). While the participants showed no significant differences in reaching distance between the *walking* mode and the no-RYSEN condition in the frontal and left direction, the participants reached further to the right side in the *walking* mode compared to the no-RYSEN condition (Table 1).

When evaluating the relationships between body weight and the differences in reaching distance, we found for reaching to the left and front small correlation coefficients varying between −0.05 and 0.25, while we found moderate to strong relationships for reaching to the right side [*balance* mode: ρ = 0.53, *p* = 0.02, 95%CI: (0.07–0.81); *walking* mode: ρ = 0.77, *p* < 0.001, 95%CI: (0.55–0.88)].

### 3.7 Questions

**Figure 6:**
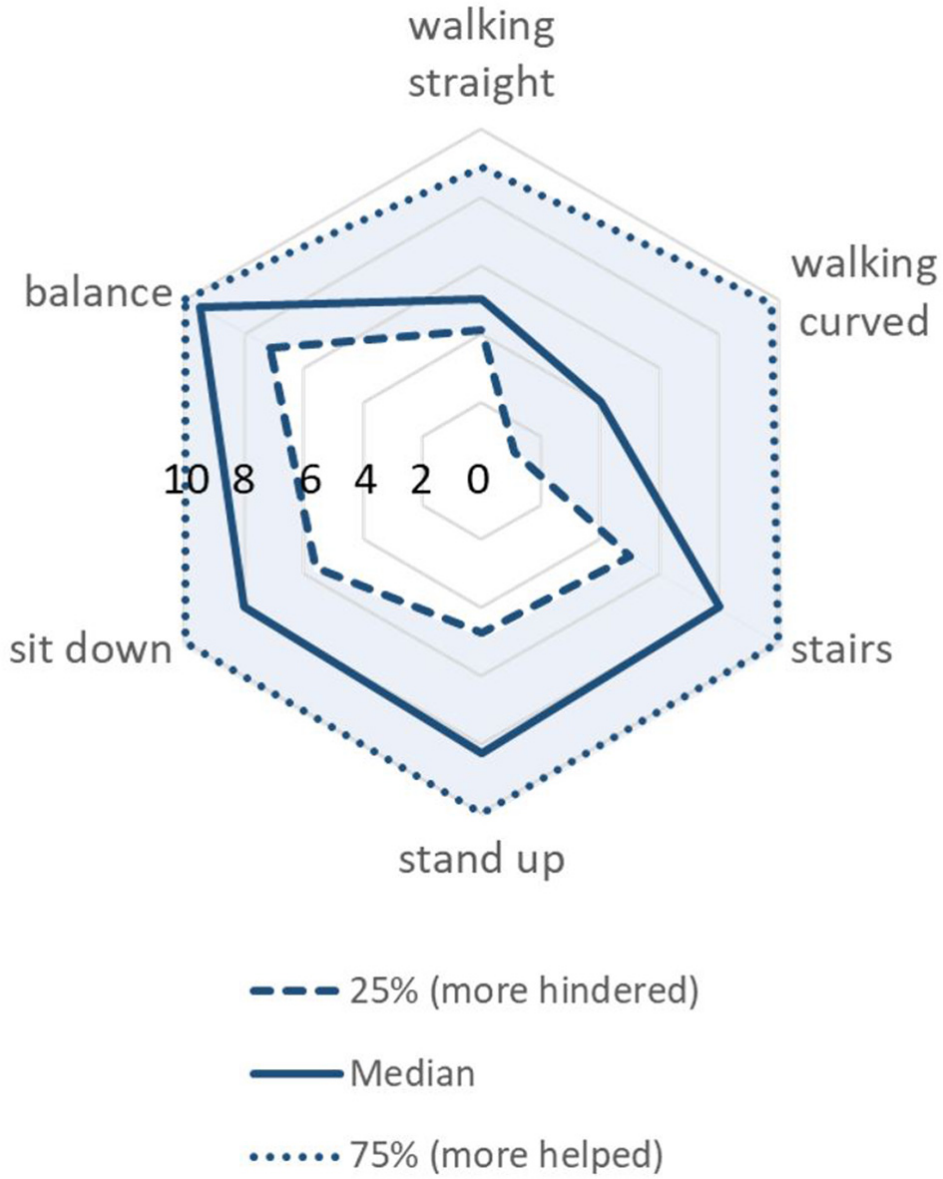
Did the RYSEN hinder or help with each task? Spider-graph showing the participants' responses, where 0 indicates “hindered a lot” and 10 “helped a lot.” The lower end of the interquartile range (IQR; i.e., 25%) is printed as a dashed line, the median (i.e., 50%) as a continuous line, and the upper end of the IQR (75%) as a dotted line. The transparent blue-colored surface reflects the IQR.

Compared to the other tasks, the participants seemed to be most hindered when walking on the curved path (Figure 6). However, the scores were not statistically different between the tasks according to Friedman's test (*p* = 0.10). Comfort of the harness was generally high at the onset of the measurement session (median: 8.5, IQR: 6.5–10) but decreased slightly but not significantly toward the end of the measurement (median: 7.25, IQR: 5.75–9.25; *p* = 0.064). Most participants reported that the measurements were a lot of fun (median: 9, IQR: 7.75–10).

## 4 Discussion

This is the first study that investigated changes in movement characteristics of various walking-related tasks induced by the RYSEN system in TDCA. Our main results were: during straight and curved walking, TDCA walked slower with shorter and more variable step lengths in the RYSEN. Meanwhile, step width (straight walking) and the deviation from a given path (curved walking) increased. When stepping up and down a 17 cm higher-level surface, the walking speed decreased while the step width increased when walking in the RYSEN. Standing up and, particularly, sitting down took longer and showed a different movement pattern with RYSEN support. Finally, TDCA reached further forward and sideways in the RYSEN *balance* mode. Interestingly, the percentile differences in various parameters (walking speed and step width during straight walking, the deviation from the predefined path and step length variability during curved walking, the step width when crossing the higher-level surface, and reaching to the right side) between the RYSEN and no-RYSEN conditions became larger in participants weighing less. Finally, questionnaire responses indicated that the RYSEN facilitated most tasks but hindered tasks like curved walking.

### 4.1 Walking straight and curved and stepping on and off a higher-level surface

According to a systematic review that included studies investigating treadmill- and overground-based body weight unloading approaches in patients and healthy individuals, approximately 30% or more body weight unloading is required to affect kinematic and spatiotemporal gait parameters (15). Five studies in that review specifically evaluated the influence of body weight support on walking speed during overground walking (16–20). According to these studies, healthy adults reduce their walking speed in conditions with body weight support compared to walking without the system (or without body weight support). Some also report a decrease in stride length (e.g., when walking overground with the KineAssist and Balance Training System (16) or Body Weight Unloading Biodex system (17)) or reduced stride time when walking with the overground robotic walker system (18). A slower speed has also been reported for individuals with stroke, who walked with a custom-made overground support system (20). Our results align with these findings because we found similar changes in gait parameters, i.e., a slower walking speed with shorter and wider steps, that occurred when walking in the RYSEN condition. We observed these changes when the participants walked on a straight or curved path and stepped on and off the higher-level surface. However, in our study, participants walked with 20% body weight unloading, which, according to the review (15), would have been too low to induce such changes. We consider the following explanations: first, an important reason can be found in Figure 4 where we plotted the participant's body weight against the changes in gait parameters between the RYSEN and no-RYSEN conditions. The plots clearly show that the deviations between the two conditions become larger as the child becomes lighter. Adolescents weighing 40 kg or more hardly show such differences, confirming the conclusion that the influence of body weight unloading on kinematic gait parameters is limited for unloading levels up to 30% (15). However, the lighter the child, the greater the deviation. Therefore, the conclusion that body weight unloading does not induce changes in kinematic and spatiotemporal gait parameters may be limited to adolescents and adults only.

Second, disturbing forces, such as the lower transparency of the RYSEN in the mediolateral direction, may be responsible for some of the findings, especially during curved walking. During straight forward walking, the TDCA experienced not much interaction in the mediolateral direction. During curved walking, though, the TDCA had to adapt to the continuously changing mediolateral resistance from the system. The resistance in mediolateral direction was reported to be more than 10 times higher than the resistance in longitudinal direction (10). Our results show that the participants deviated significantly from the curved trajectory despite walking slower. Moreover, the correlation analysis indicates that the lighter children were, again, more affected by the disturbing force. The increased resistance in the mediolateral direction may also explain why the TDCA found the RYSEN to hinder the performance of curved walking more than any other task we tested (Figure 6).

Third, another explanation could be the presence of the technology and the harness. Other studies have also mentioned that participants slowed down due to the technology itself [e.g., (16, 17, 19–21)]. During our measurements, we had the impression that participants slowed down, particularly when stepping down from the higher-level surface, because a too-fast downward movement could trigger a safety feature that temporarily increased the resistance of the RYSEN. This occurred at least once in 13 of 19 participants. This explanation would suggest that in *stairs* mode, the participants chose a more cautious gait pattern, characterized by a reduced walking speed and wider steps.

It is challenging to infer how patients might be affected by walking in the RYSEN, as they often use walking aids in daily life but not in the RYSEN, which makes direct comparisons difficult. Patients may generally walk more slowly with shorter, wider steps than their typically developing peers. Therefore, they are less likely to slow down when walking in the RYSEN. On the other hand, they have dynamic balance issues, so disturbing forces in the mediolateral direction may affect their walking more than they affect TDCA. As a consequence, therapists may initially train patients to walk in a straight line, where disturbing forces are less pronounced than on a curved path. However, these forces may be deliberately exploited in later stages of rehabilitation by including more curved walking in the RYSEN to train dynamic balance while walking. Nevertheless, therapists must consider that the effects are more pronounced in lighter patients, which may necessitate adapting training programs.

### 4.2 Stand up and sit down

Standing up and sitting down are challenging tasks that include transitioning from one stable posture to another and back again, involving movements of all body segments except the feet. Both transitions require control over complex, voluntary movement sequences. When visually comparing the trajectories of the midpoint between the acromioclavicular joint markers during the no-RYSEN condition (red trajectories in Figures 5A, C) with trajectories of young, healthy adults published in the literature (22), they appear to be consistent, providing a first validation of the task performance of the young participants during the no-RYSEN condition.

Also the ground reaction force patterns in the no-RYSEN condition seem comparable to those reported in the literature. Standing up can be divided into several phases (23, 24). When starting from a quiet sitting position, a forward momentum is generated to move the trunk forward, followed by a vertical acceleration leading to seat unloading and vertical rise. This vertical rise is initially accelerated but decelerates toward the standing position, where a stabilization phase occurs before quiet standing is achieved (23). When sitting down from a quiet standing position, there is first a transition to stooping, followed by a vertical downward movement that initially accelerates, and a long deceleration phase before the weight is being transferred to the seat. The trunk is then stabilized, and a stable sitting position is achieved (23). Indeed, the patterns of the ground reaction forces measured in our study in the no-RYSEN condition (Figures 5B, D) also seem consistent with these descriptions, validating our measurements.

It has been reported that children aged 4–6 years stand up faster than adults, which was explained by the smaller body size, i.e., the larger whole-body displacement in the adults took more time (24). Our data are in line with this, as the time needed to stand up without RYSEN correlated moderately with body height [ρ = 0.59, *p* = 0.009, 95%CI: (0.23–0.82)].

We discussed the results of standing up and sitting down in the no-RYSEN condition in detail to confirm that the young participants in this study performed these tasks in line with what is known from the literature. In contrast, when performing the tasks in the RYSEN, the trajectories (Figures 5A, C) and ground reaction forces (Figures 5B, D) deviate substantially, particularly when sitting down. Sitting down takes not only longer, the forward stooping movement is restricted. The trunk moves in an almost linear, not physiological trajectory from onset to the end of the movement.

When summarizing and generalizing these findings for clinical application to patients, we would advise against practicing standing up and sitting down in the current RYSEN *stand up* and *sit down* exercise modes. The kinematic and kinetic parameters differ meaningfully from normal task performance, particularly when sitting down. These current modes should only be used to safely transfer a patient from sitting to standing and vice versa.

### 4.3 Balance

Therapists in our clinic often use the *balance* mode to train static balance tasks or balance tasks with small internally generated perturbations, as shown in Figure 1B. However, when the TDCA performed the functional and lateral reach tasks in the RYSEN, they generated large internal perturbations as they brought their center of pressure toward the limits of stability. Several studies have examined normative values and generally show larger values for forward reach, i.e., the functional reach test, than for lateral reach in children. In 350 Indian schoolchildren aged 6–12 years, the authors reported mean values of 22.7–37.0 cm for the functional reach test and 16.3–22.5 cm for the lateral reach test (25). Similar results were reported for 280 6-to-12-year-old Turkish children (26) and 300 6-to-15-year-old Saudi children (27). These authors also reported moderate correlations with body weight and height.

In our no-RYSEN condition, the mean distance for the functional reach test was 25.8 ± 6.3 cm and for the lateral reach test 18.4 ± 4.7 cm (left) and 19.9 ± 6.9 cm (right). Thus, even though we included adolescents, the values are in good agreement with these reference values. Interestingly, while the results for the RYSEN *walking* condition were well comparable to the no-RYSEN results, the TDCA reached much further in the RYSEN *balance* exercise mode. We noticed that participants used the lateral and forward-backward support of the device and leaned into it. This may explain why most TDCA responded that the technology was helpful and not hindering (Figure 6). Instructions and corrections should be more precise and consistent in future studies because, from a therapeutic point of view, it does not make sense to lean into the technology when the therapeutic goal is to train balance actively, i.e., patients should be instructed to maintain their balance actively while pointing forward or sideways. We cannot explain why we found relationships with body weight only for pointing in the right direction. It may have to do with the footedness of the children (the right leg was the dominant leg in 12 of 19 children). In summary, although the *balance* mode offers clinically diverse opportunities to train balance, we would not recommend this mode for practicing dynamic balance tasks that require large trunk and arm movements, as also patients might simply lean into the support, as observed in the current study. For such tasks, the RYSEN *walking* mode might be preferred.

### 4.4 Methodological considerations

We did not perform a sample size calculation *a priori* because we considered this an exploratory study, as we were unaware of any comparable research that could provide meaningful insights into expected differences to inform a sample size calculation. To account for a potential low power, we also calculated effect sizes. Overall, however, the findings between the conditions were robust, with large effect sizes and statistically significant outcomes. Six parameters were not normally distributed. While we decided to present parametric outcomes for all parameters to simplify the interpretation, we also performed non-parametric analyses for these parameters, i.e., Wilcoxon signed rank tests and *r*_*ES*_. We found similar statistical outcomes and effect sizes.

We cannot rule out the possibility that some of the differences between the conditions observed in this study could be attributed to simply wearing the technology. For instance, increased step width during straight walking and navigating higher-level surfaces may be partially influenced by the position of the harness straps between the legs. However, the correlations generally suggest that these differences are not solely a result of using the technology; instead, they are influenced by the biomechanical characteristics of the technology, which appear to have a more significant effect on smaller, lighter children compared to heavier users.

The participants in the 17–25 kg weight category performed all RYSEN-related exercises with a harness weighing about 3 kg (extra weights added), leading to altered conditions due to increased inertia.

While we noticed during some pilot trials that TDCA became familiar with the system quickly, and we wanted to avoid further fatigue or loss of concentration by prolonging the familiarization period, the short familiarization may have partly contributed to the more cautious behavior of the participants when walking in the RYSEN condition.

The measurements lasted about 100 min, which could have led to fatigue, reduced concentration, or discomfort wearing the harness (due to the continuous body weight unloading in the RYSEN condition). We had included several countermeasures in our protocol, i.e., randomizing the order of the conditions, taking a break when desired, using a relatively short familiarization period, and offering a game at the end of the measurements. We think that these measures were quite successful. Although two young participants could not complete all of the balance tasks because the number of trials was too large for them, the participants generally stated at the end of the measurement session that it was enjoyable. Furthermore, although the level of comfort in wearing the harness declined slightly from the beginning to the end, it remained high.

We considered it important to have the participants wear shoes because most patients wear shoes in clinical RYSEN therapies, and for some patients, wearing (orthopedic) shoes is a prerequisite for walking. Therefore, to compare the current data with our next project in which we will investigate the applicability of the RYSEN in the actual target group, i.e., children and adolescents with gait disorders, wearing shoes was a prerequisite.

We acknowledge that placing reflective markers on the shoes and clothing (trunk) could have caused measurement inaccuracies during walking. Despite the generally high accuracy of the Vicon system, we lack information on the accuracy of the specific outcomes collected during the various walking-related tasks. However, we decided against a full clinical marker model, as placing all the markers would be impractical with the harness and time-consuming, and the protocol was already lengthy for children and adolescents (see previous limitation).

## 5 Conclusions

The RYSEN provides the exercise modes *walking, stairs, stand up, sit down*, and *balance*. Each of these modes has specific settings that therapists need to understand to personalize treatment with the RYSEN for an individual patient. Furthermore, in combination with the body weight support, these settings influence the performance of the walking-related tasks investigated in this study. The results of this study are a first step in informing therapists about the potential effects of the technology on the kinematics and spatiotemporal parameters of individuals walking in the RYSEN, particularly in children with relatively low body weight. Our next steps are to evaluate the influence of different levels of body weight support and additional horizontal forces in TDCA and the applicability of the RYSEN in the target population, i.e., children and adolescents with gait disorders.

## Data Availability

The raw data supporting the conclusions of this article will be made available by the authors, without undue reservation.

## References

[1] Boyer ER Patterson A. Gait pathology subtypes are not associated with self-reported fall frequency in children with cerebral palsy. Gait Posture. (2018) 63:189–94. doi: 10.1016/j.gaitpost.2018.05.004 29763815

[2] van Hedel HJA Aurich-Schuler T. Clinical application of rehabilitation technologies in children undergoing neurorehabilitation. In:David R Volker D, editors. Neurorehabilitation Technology. Cham, Switzerland: Springer International Publishing (2016). doi: 10.1007/978-3-319-28603-7_14

[3] van Hedel HJ Bulloni A Gut A. Prefrontal cortex and supplementary motor area activation during robot-assisted weight-supported over-ground walking in young neurological patients: a pilot fNIRS study. Front Rehabilit Sci. (2021) 2:788087. doi: 10.3389/fresc.2021.788087 36188767 PMC9397849

[4] van Hedel HJA Rosselli I Baumgartner-Ricklin S. Clinical utility of the over-ground bodyweight-supporting walking system Andago in children and youths with gait impairments. J Neuroeng Rehabil. (2021) 18:29. doi: 10.1186/s12984-021-00827-1 33557834 PMC7871598

[5] Vallery H Lutz P von Zitzewitz J Rauter G Fritschi M Everarts C . Multidirectional transparent support for overground gait training. IEEE Int Conf Rehabil Robot. (2013) 2013:6650512. doi: 10.1109/ICORR.2013.6650512 24187327

[6] Plooij M Keller U Sterke B Komi S Vallery H von Zitzewitz J. Design of RYSEN: an intrinsically safe and low-power three-dimensional overground body weight support. IEEE Robot Autom Lett. (2018) 3:2253–60. doi: 10.1109/LRA.2018.2812913

[7] Duschau-Wicke A Brunsch T Felsenstein S Vallery H Riener R. Patient-Cooperative Control: Adapting Robotic Interventions to Individual Human Capabilities. In:Dössel O Schlegel WC, editors. World Congress on Medical Physics and Biomedical Engineering. Munich, Germany. IFMBE Proceedings. Berlin, Heidelberg: Springer (2009). p. 271–4. doi: 10.1007/978-3-642-03889-1_73

[8] Lawrence DA. Stability and transparency in bilateral teleoperation. Ieee T Robot Autom. (1993) 9:624–37. doi: 10.1109/70.258054

[9] Bannwart M Bolliger M Lutz P Gantner M Rauter G. Systematic Analysis of Transparency in the Gait Rehabilitation Device the FLOAT. 14th International Conference on Control, Automation, Robotics and Vision (ICARCV 2016); 13-15th November 2016; Phuket, Thailand (2016). p. 1–6. doi: 10.1109/ICARCV.2016.7838710

[10] Plooij M Apte S Keller U Baines P Sterke B Asboth L . Neglected physical human-robot interaction may explain variable outcomes in gait neurorehabilitation research. Sci Robot. (2021) 6:eabf1888. doi: 10.1126/scirobotics.abf1888 34550719

[11] Gonzalez A Garcia L Kilby J McNair P. Robotic devices for paediatric rehabilitation: a review of design features. Biomed Eng Online. (2021) 20:89. doi: 10.1186/s12938-021-00920-5 34488777 PMC8420060

[12] Eiholzer U Fritz C Katschnig C Dinkelmann R Stephan A. Contemporary height, weight and body mass index references for children aged 0 to adulthood in Switzerland compared to the Prader reference, WHO and neighbouring countries. Ann Hum Biol. (2019) 46:437–47. doi: 10.1080/03014460.2019.1677774 31672060

[13] Duncan PW Weiner DK Chandler J Studenski S. Functional reach: a new clinical measure of balance. J Gerontol. (1990) 45:M192–7. doi: 10.1093/geronj/45.6.M192 2229941

[14] Brauer S Burns Y Galley P. Lateral reach: a clinical measure of medio-lateral postural stability. Physiother Res Int. (1999) 4:81–8. doi: 10.1002/pri.155 10444759

[15] Apte S Plooij M Vallery H. Influence of body weight unloading on human gait characteristics: a systematic review. J Neuroeng Rehabil. (2018) 15:53. doi: 10.1186/s12984-018-0380-0 29925400 PMC6011391

[16] Burgess JK Weibel GC Brown DA. Overground walking speed changes when subjected to body weight support conditions for nonimpaired and post stroke individuals. J Neuroeng Rehabil. (2010) 7:6. doi: 10.1186/1743-0003-7-6 20149244 PMC2827418

[17] Fischer AG Wolf A. Assessment of the effects of body weight unloading on overground gait biomechanical parameters. Clin Biomech. (2015) 30:454–61. doi: 10.1016/j.clinbiomech.2015.03.010 25798857

[18] Mun KR Lim SB Guo Z Yu HY. Biomechanical effects of body weight support with a novel robotic walker for over-ground gait rehabilitation. Med Biol Eng Comput. (2017) 55:315–26. doi: 10.1007/s11517-016-1515-8 27193227

[19] Patio MS Gonqalves AR Monteiro BC Santos IL Barela AMF Barela JA. Kinematic, kinetic and electromyographic characteristics of young adults walking with and without partial body weight support. Braz J Phys Ther. (2007) 11:19–25.

[20] Sousa CO Barela JA Prado-Medeiros CL Salvini TF Barela AMF. The use of body weight support on ground level: an alternative strategy for gait training of individuals with stroke. J Neuroeng Rehabil. (2009) 6:43. doi: 10.1186/1743-0003-6-43 19951435 PMC2794281

[21] Patton JL Brown D Lewis E Crombie G Santos J Makhlin A . Motility Evaluation of a Novel Overground Functional Mobility Tool for Post Stroke Rehabilitation. 2007 Ieee 10th International Conference on Rehabilitation Robotics, Vols 1 and 2. (2007). p. 1049–54. doi: 10.1109/ICORR.2007.4428553

[22] Mourey F Pozzo T Rouhier-Marcer I Didier JP A. kinematic comparison between elderly and young subjects standing up from and sitting down in a chair. Age Ageing. (1998) 27:137–46. doi: 10.1093/ageing/27.2.137 16296673

[23] Kralj A Jaeger RJ Munih M. Analysis of standing up and sitting down in humans - definitions and normative data presentation. J Biomech. (1990) 23:1123–38. doi: 10.1016/0021-9290(90)90005-N 2277047

[24] Mapaisansin P Suriyaamarit D Boonyong S. The development of sit-to-stand in typically developing children aged 4 to 12 years: movement time, trunk and lower extremity joint angles, and joint moments. Gait Posture. (2020) 76:14–21. doi: 10.1016/j.gaitpost.2019.10.030 31707306

[25] Deshmukh AA Ganesan S Tedla JS. Normal values of functional reach and lateral reach tests in Indian school children. Pediatr Phys Ther. (2011) 23:23–30. doi: 10.1097/PEP.0b013e3182099192 21304340

[26] Yuksel E Ozcan Kahraman B Nalbant A Kocak UZ Unver B. Functional reach and lateral reach tests in Turkish children. Phys Occup Ther Pediatr. (2017) 37:389–98. doi: 10.1080/01942638.2016.1205164 27462758

[27] Tedla JS Sangadala DR Gular K Reddy RS Alshahrani MS Ahmad I . Normative reference values for functional, lateral, and oblique direction reach tests in Saudi children aged six to 15 years old and psychometric properties of the oblique direction reach test. Niger J Clin Pract. (2021) 24:576–83. doi: 10.4103/njcp.njcp_102_20 33851681

